# Clinical characteristics and prognostic characterization of endometrial carcinoma: a comparative analysis of molecular typing protocols

**DOI:** 10.1186/s12885-023-10706-8

**Published:** 2023-03-14

**Authors:** Zihui Yang, Xi Yang, Xinyu Liu, Ke Ma, Yi-Ting Meng, Hong-Fang Yin, Jia Wen, Jiang-Hui Yang, Zeng Zhen, Zong-Hao Feng, Qin-Ping Liao

**Affiliations:** 1 Department of Obstetrics and Gynecology, Beijing Tsinghua Changgung Hospital, School of Clinical Medicine, Tsinghua University, No. 168 Litang Road, Changping District, Beijing, China; 2 Department of Reproductive Medicine, Shenyang 204 Hospital, Shenyang, China; 3 Department of Pathology, Beijing Tsinghua Changgung Hospital, School of Clinical Medicine, Tsinghua University, Beijing, 102218 China; 4 Institute for Intelligent Healthcare, Tsinghua University, Beijing, China

**Keywords:** TCGA typing, Endometrial carcinoma, Prognosis analysis, ProMisE typing, Application value

## Abstract

**Background:**

Endometrial carcinoma (EC) is one of the most common gynecological malignancies in China and globally, accounting for the fourth-prevalent cancer in women. Although numerous studies have confirmed prognostic value of The Cancer Genome Atlas (TCGA) molecular subgroups, it is unclear how they are combined with histological features. The main objective of this study was to compare ProMisE and TCGA classification for the rapid and accurate prediction of prognosis within EC patients, together with the provision of a revised strategy for individualized diagnosis and treatment of patients.

**Methods:**

Within this study, 70 patients with EC from Beijing Tsinghua Changgeng Hospital (affiliated to Tsinghua University) were retrospectively examined between July 2015 and December 2021. Samples were processed for determination of clinical markers, together with ProMisE and TCGA classification.

**Results:**

Comparative analysis across four TCGA types (*POLE*, Low-CN, High-CN, and MSI-H) and age, was statistically significant (χ²= 7.000, *p* = 0.029). There was no significant difference observed among the four TCGA types and FIGO stage, vascular invasion and depth of invasion, or lymph node metastasis and tumor area. There was no significant association between the expression of Vimentin, Ki-67, *PTEN*, MSH2, PAX-8, β-catenin, CD10, ER, PR, P16, *MLH1*, and *PMS2* with the four TCGA types. In addition, *p63* expression (χ²= 11.09, *p* = 0.029) and *p53* expression (χ²= 11.585, *p* = 0.005) were statistically significant. Numerous models demonstrated that patients with *POLE* mutations and low-CN had higher progression free survival (PFS) and overall survival (OS), whereas those with high-CN had lowest values. The log-rank test revealed that the survival rate of PR-positive and ER-positive patients was significantly higher (*p* < 0.001).

**Conclusion:**

Overall, these results can be of additional benefit for clinical applications, in comparison to the ProMisE classification method. In addition, PR, ER, vascular infiltration, hyperlipidemia and atherosclerosis were found to be the key factors affecting EC prognosis.

**Supplementary Information:**

The online version contains supplementary material available at 10.1186/s12885-023-10706-8.

## Highlights

TCGA molecular classification has advantages over ProMisE classification for accurate diagnosis of endometrial carcinoma.

Comparison among the four TCGA types (*POLE*, Low-CN, High-CN, and MSI-H) and age was statistically significant.

Patients with *POLE* mutations and low-CN had higher PFS and overall survival, whereas those with high-CN had the lowest overall survival periods.

**Supplementary Information:**

The online version contains supplementary material available at 10.1186/s12885-023-10706-8.

## Background

Endometrial carcinoma (EC) is one of the most common gynecological cancers, with statistics demonstrating that EC incidence and mortality rates have increased over the past decade globally. In 2022 alone, 65,950 new cases and 12,550 deaths were recorded in the United States of America (USA), highlighting the alarming increase in this under-studied malignancy [1]. In addition, data from the National Cancer Health Organization ranked EC incidence rate as being second-highest in female reproductive system malignant tumors within China, and reported a persistent increase in its incidence rate. The risk stratification of patients with EC by the national comprehensive cancer network (NCCN) is mainly based upon tumor stage / grade, and histological type [2]. According to the International Federation of Gynecology and Obstetrics (FIGO) staging system, the main treatment for EC patients with high-risk factors of recurrence is surgery, which can be combined with adjuvant chemotherapy or radiotherapy post-surgery [3]. Another promising treatment for patients with EC is immunotherapy, which targets several biological pathways by blocking immune checkpoints. Moreover, programmed cell death-1 (PD-1) and its ligands (PD-L1 or B7-H1) are targeted in EC patients with high microsatellite instability (MSI-H) or mismatch repair (MMR)-deficient tumors, which account for approximately 30% of primary EC patients [4]. However, the clinical application of immunotherapy is relatively limited, and additional clinical trials are required to confirm its therapeutic effect. Despite progress in EC treatment, the survival periods and quality-of-life of patients with EC have not been significantly improved. Patients with similar clinicopathological characteristics occasionally manifest differing disease outcomes, which could reflect the molecular heterogeneity of tumor invasion and metastasis. Therefore, novel disease-typing methods are necessary for clinicians in order to enhance accuracy in diagnosis, treatment, and prognosis.

In 2013, a multi-agency project, initiated by the National Cancer Institute and the National Human Genome Institute, analyzed bioinformatics for the cancer genome atlas (TCGA) database, and reported the genomic, transcriptomic and proteomic datasets collected from DNA sequencing, a combined DNA Methylation Reverse Phase Protein Array, together with data from microsatellite instability analysis, among 373 EC cases (306 cases of endometrioid carcinoma and 66 cases of serous / mixed carcinoma) [5]. TCGA study is the largest comprehensive genomic study of EC so far.

According to the integrated results of somatic gene mutations, based upon microsatellite instability and somatic copy number change, EC is divided into four genomic types [6]:

- The ‘super mutation’ group, which has a mutated polymerase and exonuclease domain within the *POLE* gene, is characterized by a high mutation rate [6]. A recently published study conducted by Jiang and colleagues focused upon specific immunology-based biomarker signatures for varying EC sub-types [7]. Among this study’s outcomes, it was revealed that the *POLE*-mutant EC sub-type demonstrated peak T-effector and interferon-gamma expression signatures, together with having the least innate anti-PD1 resistance expression profile among all EC sub-types. This sub-type is perceived as clinically indolent [8].
- The ‘high mutation’ group is characterized by microsatellite instability (MSI-H), typically caused by MutL homolog1 (*MLH1*) promoter methylation, high mutation rates and a few copy number changes (also known as sporadic MSI-H) [6, 9]. Interestingly, the study conducted by Bellone and colleagues revealed that, in comparison to Lynch-like MSI-H, sporadic MSI-H EC patients experienced reduced CD68 + macrophage infiltrations within tumor mass / stromal regions on administration of pembrolizumab, with such patients also experiencing reduced objective response rate (ORR), progression free survival (PFS) and overall survival (OS) timeframes in this study [9].
- The ‘low copy number’ (low-CN) group encompass most microsatellite stable grade 1 and grade 2 endometrioid carcinomas and have a low mutation rate [6]. This group is also known as having ‘no specific molecular profile (NSMP), and typically leads to prognoses that are deemed within intermediate levels between POLE-mutation and the high-CN group [10]. However, this EC group can also have widely ranging clinical manifestations – ranging from mild to highly aggressive EC within patients [10].
- The ‘high-CN group’ have extensive copy number abnormalities, low mutation rates, and recurrent *p53* mutations [6]. Among all four TCGA-stratified EC groups, the high-CN group is inherently associated with the poorest prognosis evaluations [11]. This was also reflected during the TCGA study, whereby high-CN EC group manifested across all serous carcinoma patients included in the study, together with approximately 25% of participating patients having International Federation of Gynecology and Obstetrics Grade 3 endometrioid carcinoma [12].

Recently, a novel, validated algorithm-based analytical methodology for screening potential EC cases was developed, formally known as the Proactive Molecular Risk Classifier for Endometrial Cancer (ProMisE) [13]. Such an algorithm-derived protocol utilizes datasets from p53 / mismatch repair (MMR) proteomic immunohistochemistry (IHC) together with DNA polymerase epsilon (POLE) mutation evaluation, and devised in an effort to reduce costings (and consequent patient accessibility) for POLE analyses [13]. However, although ProMisE was found to be effective and with the capacity to be rapidly adopted within medical centers, further validation-based evaluations are warranted prior to incorporation of such an algorithm-based analytical protocol for screening purposes (particularly for molecular stratifications within fertility-sparing clinical scenarios) [14, 15]. Consequently, the authors deem fit to place further focus on specific comparative analyses between the currently adopted TCGA-based molecular typing protocol and the novel algorithm-based ProMisE methodology.

Within this study, 70 EC patients admitted to Beijing Tsinghua Changgeng Hospital (affiliated to Tsinghua University) were retrospectively examined (from July 2015 to December 2021). In order to analyze epidemiological data and prognostic survival of patients with various types of EC, the collected paraffin sections were subjected to high-throughput TCGA-based molecular classification. The main aim of this study was to compare possible advantages of TCGA-based molecular classification over the algorithm-based ProMisE classification, thus performing a comprehensive evaluation and consequently providing a theoretical basis for in-depth clinical prognostic analysis for EC patients. The study also comprehended the possible molecular basis of EC treatment / research as a potential clinical guide for treatment.

## Methods

### Study participants and data collection

This study collected the clinical data of 376 patients from the Beijing Tsinghua Changgeng Hospital, suspected of having EC. The data was obtained between July 2015 to December 2021. Following data screening - based on inclusion and exclusion parameters − 70 individuals with diagnosed EC were included in the study. The selection criteria for recruited patients were based upon patients who underwent curettage or total hysterectomy at Beijing Tsinghua Changgeng Hospital, and consequently diagnosed with EC through post-surgery pathological procedures. The histological type (Grade 1–3) of endometrioid carcinoma, serous carcinoma, clear-cell carcinoma, or mixed-cell adenocarcinoma was determined for each participant. The participants did not receive any neo-adjuvant therapy or drug interference which could affect the collection of clinical information. Moreover, specimens with complete immunohistochemical and sequencing information (and without any influencing factors) formed part of study. All participants gave informed consent, while patients receiving neo-adjuvant therapy prior to surgical procedures, or patients who were suffering from other combined pelvic malignancies, were excluded from this study.

Participants with insufficient information and data, specimens with damaged immunohistochemical and sequencing information, together with participants reluctant to communicate with post-match medical staff, were all excluded from the study. The data was collected in strict compliance with standard data collection protocols.

### Sample collection and processing

DNA extraction from collected tissue samples was performed using QIAamp MinElute® DNA Extraction Kits (QIAGEN™), following manufacturer guidelines. DNA quality was assessed through Qubit® and Nanodrop®.

### Sequencing analysis

Genomic DNA (200 ng) from each sample was used in library construction. Post-fragmentation, DNA fragments were end-repaired via an A-Tailing reaction. Subsequently, T4 DNA ligase was used to link the index adapter and insert DNA fragment. Following the manufacturer’s protocol, Streptavidin Magnetic Beads (Streptavidin Sepharose®, Cytiva 17-5113-01, Sigma Aldrich™) were used for purification.

### Library amplification

The purified linkage product was amplified using 2X HotStart ReadyMix® and 10X Library Amplification Primer Mix® (KAPA Biosystems™, Massachusetts USA). Amplicons were purified using Streptavidin Magnetic Beads and the quality of the genomic DNA library was assessed on an Agilent™ 2100 Bioanalyzer®, following adjusting of concentration to 3ng/µL.

Hybridization was performed according to the standard hybridization protocol [16]. Briefly, a reaction mixture was prepared and subjected to PCR. Streptavidin Magnetic Beads (SA Beads) were used to capture and purify hybridized products, following manufacturer guidelines. The captured library prep was amplified using 2X HotStart ReadyMix® solution and 10X Library Amplification Primer Mix®, following measurement of library concentration.

### TCGA molecular classification

**Fig. 1:**
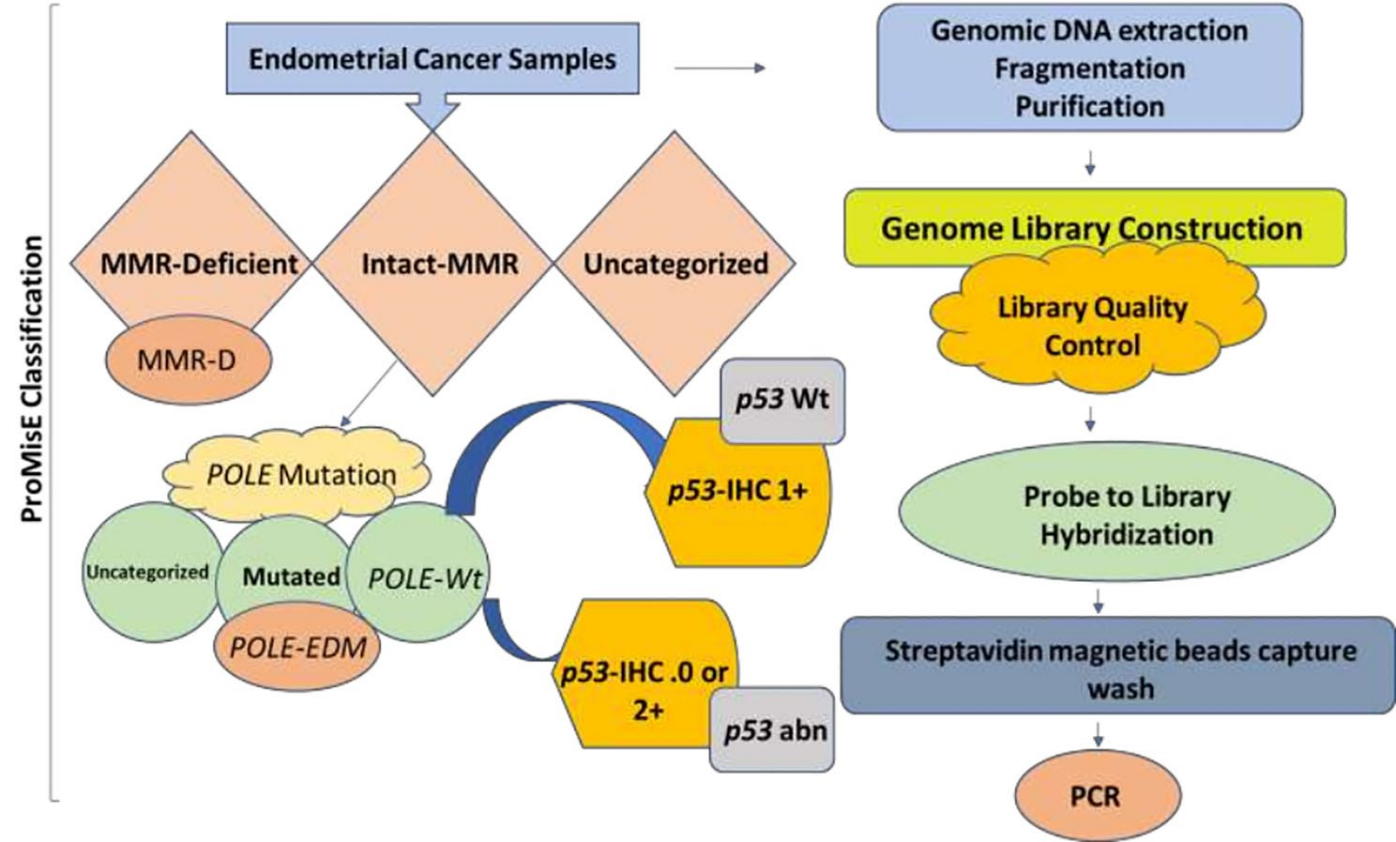
Flow chart of ProMisE and TCGA classification / sample processing

The DNA library was sequenced to cover the whole-exon region and partial-fusion mutation-related intron region of 32 genes that are closely related to EC, including four DNA mismatch repair regions, and five specific microsatellite regions. Sequencing was performed using a next generation sequencing (NGS) platform, the Illumina™ HiSeq 2000® Sequencing System, and the reference genome GRCh37 - hg19. Analysis was performed using the standard TCGA Molecular Typing Method, by categorizing the samples into four groups: *POLE* super-mutation, MSI-H, low-CN, and high-CN. The specific classification process included the detection of mutation status for *POLE* gene. The presence of mutations within the *POLE* gene was classified as *POLE* super-mutation. MSI was sequenced at four gene loci, including MLH1, MSH2, MSH6, and PMS2 within wild-type sample for *POLE* gene. The presence of mutations in one of these genes was defined to be MSI-H. The presence of *p53* gene mutations was regarded as high-CN, while the absence of mutations was classified as low-CN (Fig. 1).

### Immunohistochemistry

#### Sample preparation

Fresh, dissected tissues (< 3 mm thick) were collected and fixed with 2% paraformaldehyde. Each dissected tissue was embedded in paraffin blocks, which were sliced into 5–8 μm segments. Samples were stained using a standard staining procedure and stored at room temperature until further analysis. Immunohistochemical markers, together with the associated detection criteria, are described in Table S1.

### ProMisE classification

Molecular classification involving the ProMisE method included the detection of MMR protein/s by immunohistochemistry, in order to identify patients and screen for Lynch syndrome (Fig. 1). The results directed the patients onto either surgical or treatment decisions. The polymerase enzyme for the individual tumor was evaluated- ɛ (*POLE*) through exonuclease domain mutation (EDM) sequencing results. Finally, protein 53 (*p53*) was evaluated through immunohistochemistry, resulting in four subgroups of mutations that included MMR-d, *POLE*, *p53* wild-type (wt), and *p53* null / missense mutation (abn).

### Statistical analysis

Statistical analysis was performed using a two-sided t-test, and statistical significance was determined by α = 0.05, and *p* ≤ 0.05. All datasets were expressed as the mean standard deviation (x ± s). Two independent sample t-tests were used for comparison between different groups, and IBM™ SPSS® statistics 26. 0 (Chicago, USA) was used for statistical analyses.

## Results

### Baseline clinical characteristics

Patient age ranged between 38 and 83 (mean age 60 ± 1.41) years; three with *POLE* super-mutations, nine with MSI-H, 45 with low-CN, and 13 with high-CN. Patient age within *POLE* super-mutation, MSI-H, low-CN and high-CN groups ranged between 50 and 79 (mean age 63.00 ± 14.73) years, 45–82 (mean age 61.20 ± 12.13) years, 40–80 (60.02 ± 9.59) years, and 38–83 (62.17 ± 12.14) years, respectively. Patient age among all four groups was significantly different (χ²= 7.000, *p* = 0.029, Table 1).

**Table 1:** Comparison of differing age groups in endometrial cancer (EC) patients, with four TCGA molecular types

Category	Total number of cases	Pole mutant	MSI-H type	Low CN type	High CN type	χ² value	*P* value
Age (years)	70					7.000	0.029
≤ 60	34	2	5	23	4		
> 60	36	1	4	22	9		

Menopausal status, clinical manifestations, complications, and other data for each patient (n = 70) is shown in Table S2. The youngest postmenopausal patient was 39 years old and the oldest was 60 years old (mean age 51.62 ± 4.26 years). The age of post-menopausal patients within MSI-H, low-CN and high-CN groups ranged from 51 to 57 (mean age 54.17 ± 2.48) years, 39–60 (mean age 50.76 ± 4.28) years and 45–60 (mean age 52.33 ± 4.58) years, respectively. Menopausal status of patients was not significantly different among the four groups (χ²= 1.987, *p* = 0.624). There was no significant difference within clinical manifestations, including abnormal uterine bleeding, abnormal imaging manifestations, vaginal drainage, cervicitis, and hyperlipidemia observed among EC patients with differing TCGA groups (*p* > 0.05). The majority of patients with EC had complications, including hypertension, diabetes, atherosclerosis, uterine leiomyoma, latent syphilis, and human papillomavirus. No significant difference between EC patients and the differing TCGA groups was observed (*p* > 0.05). Any significant difference between patient body mass index (BMI) (< 28 and ≥ 28), treatment route (laparotomy versus laparoscopy), therapy type (adjuvant versus chemotherapy), carcinoembryonic antigen (CEA) levels, and levels of cancer antigens (i.e., 19 − 9, 15 − 3 and 12 − 5 between the four differing TCGA groups) was not observed. The CEA level was increased in one patient (2.44%) who was in the low-CN group. There was no significant difference in CEA indexes between the two groups (χ²= 3.055, *p* = 1.000). Similarly, cancer antigens CA-1-5, CA-15-3 and CA-19-0 demonstrated increased levels in several patients. However, there was no difference in CEA indexes between the groups of patients with elevated and normal CA levels. The specific analysis of clinical baseline characteristic data is shown in Table S2.

### Pathological data

#### Pathological classification

Based on pathological classification, the 70 samples were divided into type I EC (n = 64) and type II EC (n = 6), according to morphological manifestations and immunohistochemical staining results. The type II EC group included four cases of serous carcinoma, one case of clear cell carcinoma, and one case of endometrial serous papillary carcinoma. The TCGA classification of Type I EC and Type II EC groups is shown in Table S3 and indicated statistical significance between the two groups, based upon pathological classification.

### Pathological grading

Based on the TCGA classification of EC, the 70 cases were divided into G1 (n = 24), G2 (n = 36) and G3 (n = 9) grade. One case did not belong to any category and was referred to as an ‘unknown grade’. EC samples were categorized as either *POLE* super-mutation (n = 1), MSI-H (n = 8), low-CN (n = 43), or high-CN (n = 8). G3 cases were categorized into *POLE* super-mutation (n = 1), MSI-H (n = 1), low-CN (n = 2), and high-CN (n = 5). Statistically significant differences in pathological grades between the two groups (G1 ~ G2, and G3) were observed (χ²= 11.098, and *p* = 0.006, Table S4).

### Pathological stage

FIGO staging was used to determine the pathological stage of EC within participants (Table 2). One case could not be categorized and was regarded as an ‘unknown’. There was no significant difference in FIGO stage and the four differing TCGA molecular types in patients with EC (χ²= 2.947, *p* = 0.380).

**Table 2:** Comparison of differing pathological stages and immunohistochemical markers in endometrial cancer (EC) patients, with four TCGA molecular types

Category	Total Number of Cases	Pole Mutant	MSI-H Type	Low CN Type	High CN Type	Χ² Value	*P* Value
**FIGO Staging**	69					2.768	0.383
I	58	3	8	35	12		
II-IV	11	0	0	10	1		
**PTEN expression**	65					1.844	0.613
Positive	9	0	2	5	2		
Negative	56	3	6	38	9		
**P53 Expression**	65					11.09	0.029
Positive	13	0	2	4	7		
Negative	52	3	6	39	4		
**MLH1 Expression**	65					3.052	0.029
Positive	52	2	5	35	10		
Negative	13	1	3	8	1		
**MSH2 Expression**	65					2.720	1.000
Positive	64	3	8	42	11		
Negative	2	0	0	1	0		
**PMS2 Expression**	65					2.796	0.410
Positive	51	2	5	34	10		
Negative	14	1	3	9	1		
**MSH6 Expression**	65					-	-
Positive	65	3	8	43	11		
**Pax-8 Expression**	65					1.915	0.646
Positive	20	1	4	12	3		
Negative	45	2	4	31	8		
**β- Catenin Expression**	65					0.809	0.895
Positive	43	2	6	27	8		
Negative	22	1	2	16	3		
**CD10 Expression**	65					0.765	1.000
Positive	16	0	2	11	3		
Negative	49	3	6	32	8		
**ER Expression**	65					2.447	0.513
Positive	57	2	8	37	10		
Negative	8	1	0	6	1		
**PR Expression**	65					4.114	0.171
Positive	56	3	8	38	8		
Negative	9	1	0	5	3		
**P16 Expression**	65					4.546	0.193
Positive	25	3	3	15	4		
Negative	40	0	5	28	7		
**P63 Expression**	65					11.585	0.005
Positive	8	1	4	2	1		
Negative	57	2	4	41	10		

### Immunohistochemistry

Staining results of markers related to the pathological occurrence and EC development were analyzed. These markers included Vimentin, Ki-67, PTCN-2, *p53*, MLH1, MSH2, PMS2, MSH6, Pax-8, β- Catenin, CD10, *p16*, ER, PR, *p63*. The number of samples being positive for each marker, together with positivity rate and corresponding TCGA classification for each sample, is depicted in Table 2. There were no significant variations in Vimentin, Ki-67, PTCN-2, MSH2, PMS2, MSH6, Pax-8, β- Catenin, CD10, ER, and p16 indexes of patients with differing TCGA typing. However, there was a significant difference observed between p53, MLH1, and *p63* indexes of patients with differing TCGA typing (Table 3).

**Table 3:** Comparison of differing immunohistochemical expression for various tumor markers in endometrial cancer patients, with four TCGA molecular types

Category	Total Number of Cases	Pole Mutant	MSI-H Type	Low CN Type	High CN Type	Χ² Value	*P* Value
**Vimentin Expression**	65					1.908	0.559
Positive	53	2	7	36	8		
Negative	12	1	1	7	3		
**Ki-67 Expression**	65					1.105	0.837
Positive	46	2	6	29	9		
Negative	19	1	2	14	2		

### Histopathology

The tumor area, vascular infiltration, depth of infiltration, as well as the presence of myometrial invasion, was determined for each case. Individual patient tumor size was measured and classified into TCGA groups (Table S5). There was no significant difference between tumor size and the TCGA groups within EC patients(χ²= 0.536, *p* = 1.000). Each patient was examined for lymph node metastasis and classified into TCGA groups (Table S6). There was no significant difference in lymph node metastasis with TCGA groups among EC patients (χ²= 1.271, *p* = 1.000).

Among all analyzed samples, 13 patients had vascular infiltration. The pathological data for 12 patients were not displayed or obtained. There was no significant difference between the presence of vascular infiltration and TCGA groups among EC patients (χ²= 1.499, *p* = 0.742). Myometrial invasion was detected within 46 patients. Seventeen patients had myometrial invasion (> 50%). Two cases had unknown information. There was no significant difference in lymph node metastasis between the four TCGA groups of EC patients (χ²= 3.715, *p* = 0.277).

### Prognosis analysis within EC patients

#### Pathological classification

**Fig. 2:**
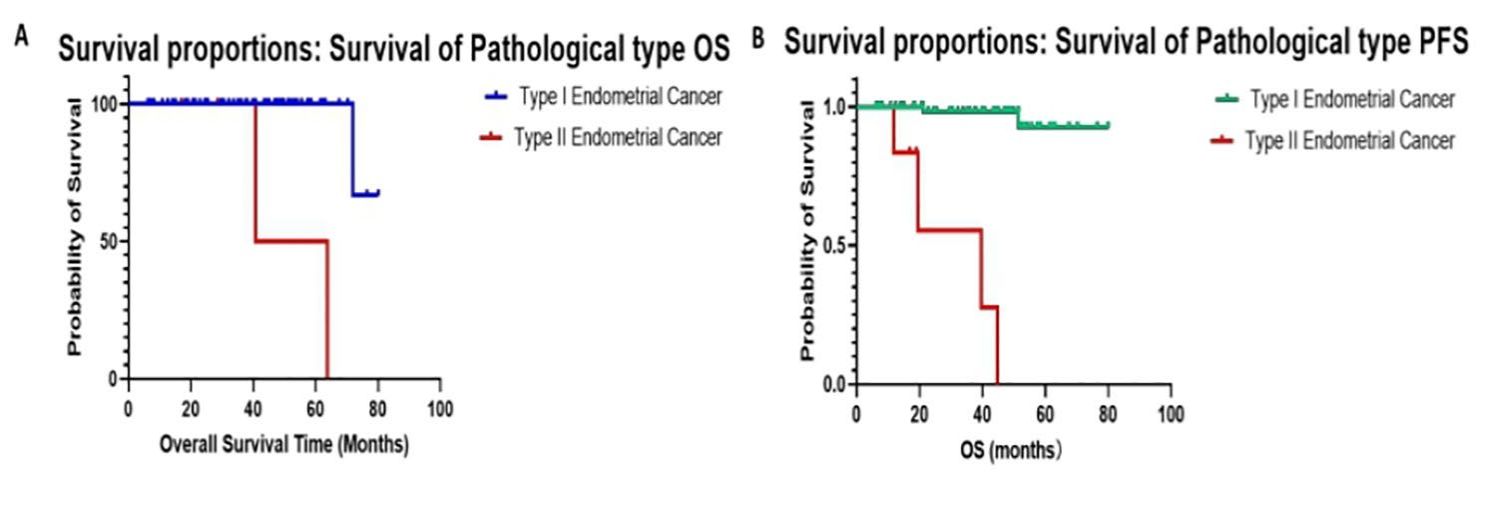
Overall survival curve (*p* < 0.001) (A) and progression free survival curve (*p* < 0.001) (B) for patients with type I and type II endometrial cancer

Among the 70 patients, there were 64 cases of EC, four cases of serous carcinoma, one case of clear cell carcinoma, and one case of serous papillary carcinoma of the endometrium, which were all classified as type II endometrial carcinoma. Three people died of the disease (4.29%), including one case of endometrioid carcinoma, one case of clear cell carcinoma, and one case of endometrial serous papillary carcinoma. There were 63 cases of progression-free patients (92.20%), and seven cases of recurrence (6.20%), including three cases of endometrioid carcinoma, two cases of serous carcinoma, one case of clear cell carcinoma, and one case of serous papillary carcinoma of endometrium. There were significant differences in the number of mortalities and recurrences due to the disease. Excluding non-diseased mortalities or life-accidents, the four molecular types were compared by log-rank method. The overall survival (OS) of type I EC and type II EC were 98.4% and 66.7% respectively (*p* < 0.001). In addition, there was a significant difference between type I endometrial cancer and type II endometrial cancer. The progression free survival (PFS) of type II endometrial carcinoma were 96.9% and 33.3%, respectively (*p* < 0.001). The survival curve for type II EC was significantly lower, and prognosis was worse than within type I EC (Fig. 2A, B).

### TCGA molecular typing of genes with non-pathogenic functions

Prognostic analysis was based upon 65 samples, as several samples were not found during immunohistochemistry. The median follow-up time was 1234 days, with no loss of follow-up for all patients. During this time, three patients died (4.62%); two in the high-CN group (66.67%) and one in the low-CN group (33.33%). There were six patients with recurrence (9.23%); one in the MSI-H group (16.67%), one in the low-CN group (16.67%), and four in the high-CN group (66.67%). Excluding non-diseased mortality or life-accidents, the four molecular typing groups were compared through log-rank method. The survival curve was obtained by Kaplan Meier method. The overall survival (OS) of patients with *POLE* super-mutation, MSI-H, low-CN, and high-CN were 100%, 93.3%, 100%, and 80.0%, respectively. There was a significant difference between the four typing groups (*p* = 0.005). The progression free survival (PFS) of patients with *POLE* super-mutation, MSI-H, low-CN and high-CN were 92.3%, 86.7%, 100% and 70.0%, respectively (*p* = 0.007). Patients within *POLE* mutation and low-CN groups had higher PFS and OS, while patients in the high-CN group had the lowest OS timeframes (Figure S1, S2).

### TCGA molecular typing

The four molecular typing groups were compared through log-rank method. The OS of patients with *POLE* super-mutation, MSI-H, low-CN and high-CN were 100%, 100%, 97.60% and 84.60%, respectively. There was no significant difference between the four TCGA groups (*p* = 0.380). The PFS of *POLE* mutant, MSI-H, low-CN, and high-CN were 100%, 87.50%, 97.50% and 69.20% respectively, and a significant difference among the four groups (*p* < 0.001) was observed. Patients within *POLE* super-mutation and low-CN groups had higher PFS and OS, while patients in the high-CN group had lowest PFS and OS timeframes (Figure S3, S4).

### ProMisE typing

**Fig. 3:**
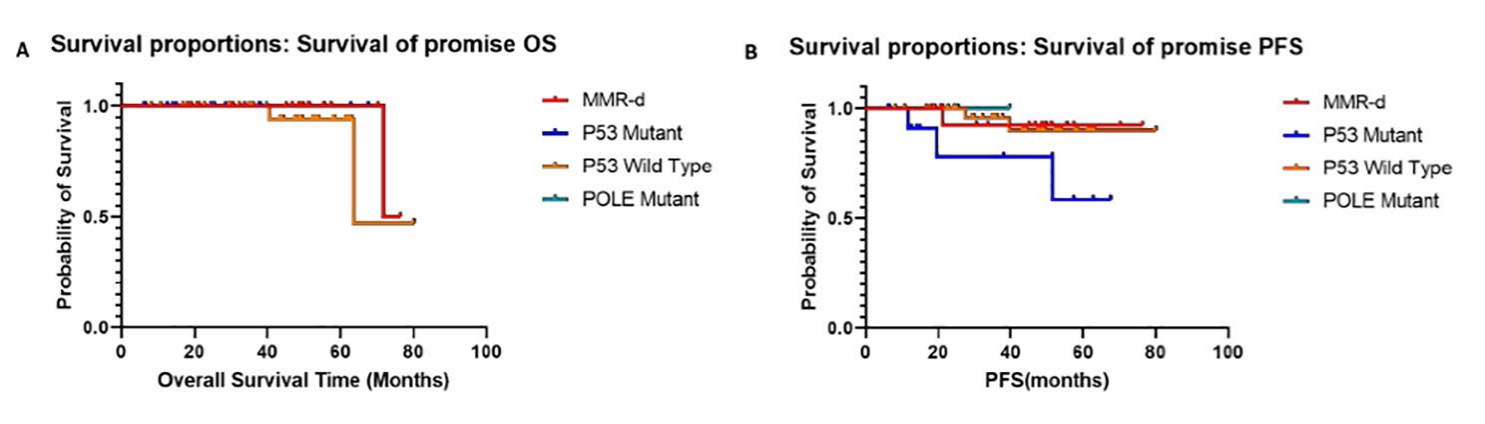
Overall survival curve (*p* = 0.394) (A) and progression free survival curve (*p* = 0.010) (B) for patients with endometrial cancer, as classified by ProMisE.

Among the 65 patients with TCGA molecular typing, 18 cases were ranked in the MMR-D group (23.08%), two cases in the *POLE* mutation group (4.62%), 11 cases in the *p53*abn group (18.46%), and 34 cases in the *p53*wt group (53.85%). Overall, three people died of the disease; one from the MMR-D group (6.20%), and two from the *p53*abn group (6.20%). Six patients experienced recurrence (9.23%): one from the MMR-D group (16.67%), four from the *p53*abn group (66.67%) and one from the *p53*wt group (16.67%). Excluding non-diseased mortality or life-accidents, the four molecular types were compared through log-rank method. The OS for MMR-D, *POLE* mutation, *p53*abn and *p53*wt groups were 94.4%, 100.0%, 81.8% and 100.00%, respectively. There was no significant difference among the four groups (*p* = 0.394). In addition, the PFS for MMR-D, *POLE* mutation, p53abn, and p53wt groups were 94.4%, 100.00%, 63.60% and 97.10%, respectively. Differences among the four groups was statistically significant (*p* = 0.010). The patients in *POLE* mutation and *p53*wt groups had higher PFS and OS, while EC patients in the *p53*abn group had the lowest survival rate (Fig. 3).

### Correlation analysis of associated factors

Three of the 70 patients died, and the median follow-up time was 41 months. The 3-year, 5-year and 6-year survival rate was 100.00%, 98.57%, and 95.71%, respectively. In terms of recurrence in patients with EC, six of the 70 patients relapsed, with a 1-year, 3-year and 5-year recurrence rate of 1.43%, 5.71% and 8.33%, respectively.

**Fig. 4:**
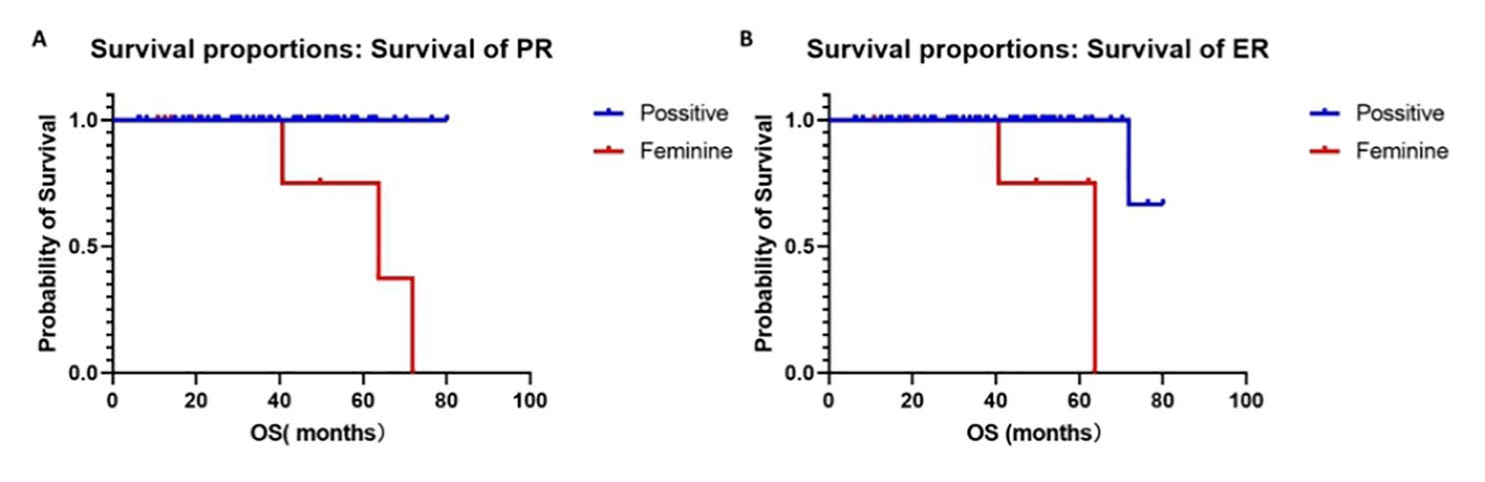
Overall survival curve of endometrial cancer patients positive for progesterone receptor (*p* = 0.003) (A), and estrogen receptor (*p* = 0.001) (B)

The log-rank test was used to analyze prognostic factors (Fig. 4A-B). The results showed that the survival rate of progesterone receptor (PR) positive in patients was significantly higher than for PR negative patients (*p* = 0.003). Similarly, the survival rate of estrogen receptor (ER) positive patients was significantly higher than for ER negative patients (*p* = 0.001).

**Fig. 5:**
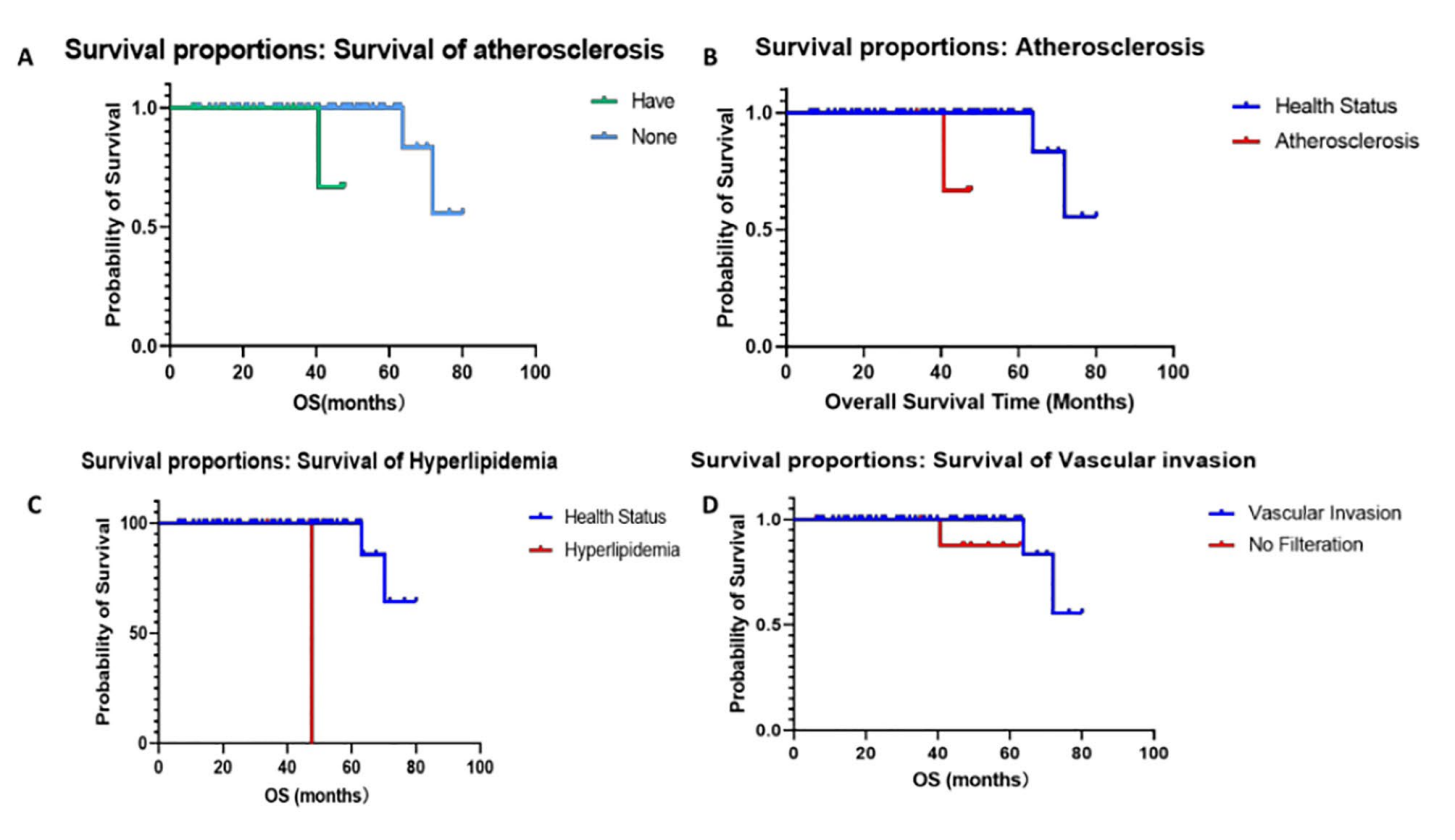
Survival proportions of patients with atherosclerosis (A), comparison of health status of patients with atherosclerosis (*p* < 0.001) (B), survival of patients with hyperlipidemia (*p* = 0.001) (C), and survival proportions of patients with vascular invasion (*p* = 0.011) (D)

The survival rate of patients with vascular infiltration was lower than that of patients without vascular infiltration (*p* = 0.011), while survival rate of patients with hyperlipidemia was lower than that of patients without this disease (*p* = 0.001). The survival rate of patients with atherosclerosis was significantly lower than that of non-atherosclerotic patients (*p* < 0.001, Fig. 5A-D).

FIGO stage (*p* = 1.000), age (*p* = 0.251), BMI index (*p* = 0.190), family history of tumor (*p* = 0.369), degree of differentiation (*p* = 0.369), surgical approach (*p* = 0.982), degree of myometrial infiltration (*p* = 0.140), tumor size diameter (*p* = 0.154), menopausal status (*p* = 0.184), radiotherapy (*p* = 0.868), chemotherapy (*p* = 0.426), imaging findings (*p* = 0.405), uterine leiomyoma (*p* = 0.881), diabetes mellitus (*p* = 0.874), high blood pressure (*p* = 0.793), abnormal uterine bleeding (*p* = 1.000), human papillomavirus (*p* = 1.000), cervicitis (*p* = 0.766), vaginal drainage (*p* = 1.000), negative syphilis (*p* = 0.306), CA153 (*p* = 1.000), CA125 (*p* = 0.480), P16 (*p* = 0.298),Vimentin (*p* = 0.592), β- Catenin (*p* = 0.251), Pax-8 (*p* = 0.370), *p53* (*p* = 0.692), Ki-67 (*p* = 0.572), and PTEN (*p* = 0.584) all had no significant correlation with prognosis.

## Discussion

The clinical value of prognostic / predictive tools can hardly be underestimated for ensuring the accurate potential disease progression within individual patients, and thus allowing for the implementation of bespoke therapeutic strategies for maximizing successful outcomes (including extended OS and PFS timeframes) within such patients. Such predictive tools are invariable more important for specific tumors such as EC, whereby the presence of multiple risk factors can affect EC manifestation and clinical aggressiveness [17–25]. Moreover, the ability to characterize tumors on a genomic level can have potentially crucial clinical value, as – with such knowledge – oncologists and clinical staff will have enhanced awareness and clinical foresight in order to perform more informed and bespoke therapeutic and patient management decisions, in order to ensure successful treatments. Consequently, on focusing upon EC, accurate genomic characterization concerning EC molecular typing / groupings can pivot therapeutic outcomes within patients towards a more favorable clinical endpoint. One milestone achievement was the EC molecular typing prediction tool developed by the Cancer Genome Atlas Research Network in 2013, whereby a total of 373 EC samples were mapped in an integrated manner for proteomic / transcriptomic / genomic expression profile identification [26]. Such great research efforts by the TCGA research network led to the above-described gold-standard EC molecular typing classification, consisting of four major EC molecular-type subgroups, currently still utilized by oncologists on a global scale [26].

Although the prognostic value of TCGA subgroups has been confirmed within many studies, it is unclear how they are combined with histological features, such as tumor grade and histological type. Moreover, the prognostic factors in pathology are always disturbed in clinical diagnosis and evaluation, including repeatability of histological and FIGO classifications. There is often overlap between histological subtypes and grading, which complicates clinical decision-making. Therefore, the diagnostic consistency between observers is still not ideal, especially within high-grade histotypes and frozen paraffin specimens [27]. In addition to TCGA, ProMisE typing has shown its clinical significance in the treatment and diagnosis of EC patients [28]. ProMisE divides EC patients into four prognostic groups: *POLE* mutation (*POLE* MT), mismatch repair defect (mmr-d), *p53* abnormal (*p53* abn), and *p53* wild type (*p53* wt)[28]. The *POLE* MT group includes EC with the best prognosis and the highest mutation load. This group is characterized by polymerase ε (*POLE*) and is the only group that can be fully identified by sequencing. The prognosis of the MMR-d group is moderate, the mutation load is high, and the microsatellite is unstable. This group can be identified by the defective immunohistochemical expression of the mismatch repair protein (MMR). The *p53* abn group has the worst prognosis, low mutation load, a high rate of somatic copy number variation and a high mutation rate of TP53. This group can be identified by the abnormal immunohistochemical expression of *p53*. The *p53* wt group has a moderate prognosis, low mutation load, low rate of somatic copy number variation, and no molecular characteristics. This group is typically identified by excluding molecular characteristics of other groups [29].

Consequently, the TCGA and ProMisE classifications were comparatively analyzed across a cohort of 70 EC patients, in order to evaluate the optimal classification system for molecular-type-based EC patient classification.

In this study, the only variable that reached statistical significance when comparing the four TCGA subgroups was age. Patients > 60 years also tended to have high-CN and less *POLE* super-mutants. Following the TCGA-based classification, this study observed a significant difference between type I and type II EC in pathological classification (χ²= 9.437, *p* = 0.013) among EC patients. There was a significant difference in pathological grade between the G1 / G2 and G3 group (χ²= 11.098, *p* = 0.006). Moreover, IHC analysis demonstrated insignificant dysregulated expression of vimentin, Ki-67, PTEN, MSH2, Pax-8, and β- catenin. There was no significant difference between the four subgroups of TCGA typing and dysregulated expression of CD10, ER, PR, and p16. Only p63 expression (χ²= 11.585, *p* = 0.005), and p53 expression (χ²= 11.090, *p* = 0.029) were significant. Additionally, MSH6 was upregulated across all patients, possibly suggesting that *p63, p53* and MSH6 proteins within the MSI family can play important roles within EC. Similar to such results, MSH6 is a high-risk factor affecting patient prognosis, with such expression levels being linked to patient clinicopathological parameters [30]. Being a crucial gene, *p53* plays a role within many tumors, providing that wild-type TP53 can activate natural cellular immune response of cells, while mutant TP53 can lead to the immune-escape of tumor cells by negatively regulating cellular natural-immune-signaling, thus promoting the recurrence and metastasis of tumors [31]. MSI-H mutations are common in EC, and the frequency of benign endometrial lesions is often high. Within this study, MSI-H mutations in EC patients were associated with increased tumor grade, severe myometrial infiltration, together with increased clinical stage, as expected. In this study, the only variable that reached statistical significance when comparing the four TCGA subgroups was age. Patients > 60 years also tended to have high-CN and less *POLE* super-mutants.

Comparison of high-throughput sequencing results indicated that *p53* immunostaining had elevated accuracy in predicting TP53 mutations within EC, with a consistency of 67.14% (the accuracy of MMR and MSI-H mutations was 46.88%). Such observations suggest that there exists a potential correlation between EC patients and pathological features, which needs to be discussed further. It is reported that somatic mutations within the *POLE* gene are found in 6–10% of EC patients, and are associated with improving relapse-free survival and germline mutations in patients with grade 3 endometrioid EC [29][36]. Within this study, *POLE* mutations accounted for 4.82% of all study participants. Tumor suppressor phosphatase and tensin homologue (PTEN) is a phospholipid phosphatase, which counteracts the activity of phosphatidylinositol 3-kinase by dephosphorylating phosphatidylinositol 3-kinase by phosphorylating the D3 position of the inositol ring of phosphatidylinositol. Similarly, TP53 acts in the case of DNA damage and inhibits the cell cycle in the G1 / S phase, thereby activating the repair mechanism. The dysfunction of p53 in malignant tumors is mainly due to the inactivation of the p53 protein through binding protein or TP53 mutation.

Within type I EC, TP53 was found to be upregulated, together with PTEN downregulation within higher EC grades [32]. Consequently, the immune expression evaluation of TP53 is helpful to the diagnosis and treatment of various EC types of EC. Secondly, TP53 is a prognostic biomarker for this type of tumor, with TP53 genomic mutations accounting for 7.88% of participants in this study. Pathological classification was based upon TCGA typing, where molecular types were compared through log-rank method. The survival curve using Kaplan Meier method demonstrated that OS for type I EC was higher than that of type II EC. Overall, survival rate of patients with type II EC was significantly lower, together with worse prognosis when compared to patients having type I EC. Finally, ProMisE typing was performed to analyze the EC patient prognoses. There was no significant difference in OS across all four groups (*p* = 0.585). However, PFS among the four groups was significantly different (*p* = 0.012). The PFS time and prognosis for the p53wt group were significantly lower than for MMR-d and *POLE* mutation groups. In addition, within ProMisE typing, it was found that results obtained by high-throughput sequencing of MMR IHC exhibited *MLH1* and *PMS2* downregulation. This phenomenon was previously linked to *MLH1* promoter hypermethylation [37–38]. Another possible explanation for differing results of IHC and high-throughput sequencing is that existed an abundance of normal nuclei within selected EC tissue, with mutant MSI alleles not being detected through high-throughput sequencing.

Following comparative analyses for TCGA and ProMisE molecular typing protocol performance in assessing the investigated EC cohort within this study, the TCGA revealed to have increased resolution and robustness in predicting OS and PFS timeframes, in comparison to the ProMisE molecular typing protocol. The added resolution for TCGA molecular protocol lies in the premise that also additional risk factors are taken into consideration when performing predictive evaluations for EC patients with the TCGA protocol.

This study does have several limitations. Firstly, the patient cohort of 70 EC cases admitted to our institute, across a period of 6.5 years, is relatively small and can possibly not be fully representative for study outcomes. Secondly, the lack of an external validation cohort in our study is due to the fact that the use of ProMisE typing is still not widespread among most clinicians in China. Importantly, our study was conducted as a retrospective study, highlighted by its clinical relevance analysis in EC patients. In the future, we will supplement ProMisE subtyping analysis with large samples based on Chinese population. However, a follow-up study utilizing a second patient cohort is contemplated, in order to perform temporal validation of such study results. Thirdly, during the NGS investigation, a large number of samples were still not identified correctly due to the inherent challenges involved within immunostaining biopsy specimens using prolonged fixation time and the presence of differing tumor cell structures (extracted from IHC and DNA-extracted sections). This could also be linked to the difficulty of amplicon deep-sequencing method applied to all samples, in order to detect stop-acquisition / splicing mutations and large deletions / insertions, especially within endometrial biopsy samples. Finally, possible errors in establishing the difference between MMR and MSI could be due to the presence of too many normal nuclei (normal endometrium and a large number of tumor infiltrating lymphocytes) in order to detect microsatellite unstable mutant alleles through NGS.

In essence, the main outcomes and inferences from this study are outlined below, namely:

A) TCGA molecular classification has advantages over ProMisE classification for accurate diagnosis of endometrial carcinoma.
B) Comparison among the four TCGA types (POLE, Low-CN, High-CN, and MSI-H) and age was statistically significant.
C) Patients with POLE mutations and low-CN had increased PFS and OS, whereas those with high-CN had the lowest overall survival periods.

## Conclusion

Presently, diagnostic and classification protocols for EC are still evolving. Modernized, rapid and accurate molecular diagnostic features certainly aid in the integration of molecular subtype diagnosis within clinical practice. Moreover, molecular subtypes are of undoubted predictive importance. Hence, it is particularly important to conduct auxiliary research on *POLE* mutation, MMR / MSI and abnormal p53 expression. The advantage of TCGA molecular subtype diagnosis method is that it can generate all datasets required by molecular subtypes and other genomic information apart from molecular subtypes, including β- Catenin mutation status, tumor mutation load (TMB) and other parameters, which can guide treatment. The survival analysis demonstrated that TCGA model is more beneficial than ProMisE typing when predicting patient prognosis. Nevertheless, ProMisE typing is less costly, easier, and provides rapid data access. The combination of these two methods provides additional unique information for the diagnosis and prognosis of prose subtypes, though are more valuable in the molecular classification of prose subtypes. Finally, TCGA molecular typing for EC has feasibility and application value within a clinical setting.

## Electronic supplementary material

Below is the link to the electronic supplementary material.

Supplementary Material 1: Table S1: Panel of immunohistochemical markers and associated diagnostic criteria

## Data Availability

The datasets generated and/or analyzed during the current study are available in the TCGA repository and are also available as supplementary material.

## Declarations

Consent for Publish

NA.
