# Supplementary material for: Clinical characteristics and prognostic characterization of endometrial carcinoma: a comparative analysis of molecular typing protocols
Source: BMC Cancer. 2023 Mar 14;23:243. doi: 10.1186/s12885-023-10706-8 (PMC10015692; doi:10.1186/s12885-023-10706-8)
Supplement: Supplementary file 1 — Supplementary Material 1: Table S1: Panel of immunohistochemical markers and associated diagnostic criteria [file 12885_2023_10706_MOESM1_ESM.docx]

Table S1: Panel of immunohistochemical markers and associated diagnostic criteria

| Immunohistochemical Marker | % of tumor cells stained and considered positive | Diagnostic criteria/observations | Cellular location of staining |
| --- | --- | --- | --- |
| Vimentin | >10% |  | Cytoplasm of tumor cells |
| Ki-67 | >20% |  |  |
| PTEN | >10% | A total of 100 endometrial cancer cells in 5 visual fields were selected for counting | Cytoplasm of tumor cells |
| P53 | 70% (p53abn)  <10% negative (p53wt) | Count 5 visual fields under 400 X magnification with 200 tumor cells in each visual field |  |
| MLH1, MSH2, PMS2, and MSH6 (MMR proteins) | 10% |  |  |
| Pax-8 | ≥50% |  |  |
| β- Catenin | 10% | Reference staining intensity divided into strong, medium, weak, and negative | Nucleus |
| CD10 | 10-50% deemed as low expression, and > 50% deemed as high expression. | mean percentages of positive cells in tumor (epithelial component) and / or stromal (interstitial component around epithelial cells) cells were calculated | Cytoplasm and / or cellular membrane |
| Estrogen Receptor (ER | > 1-10% |  | Nucleus |
| Progesterone Receptor (PR | > 1-10% |  | Nucleus |
| P16 | 70% (p16abn)  <10% positive cells (p16wt) | Count 5 visual fields under 400 X magnification with 200 tumor cells in each visual field |  |
| P63 | >50% |  | Cytoplasm |

Table S2. Comparison of menopausal status, clinical manifestations, complications, and data for other clinical baseline characteristics in patients with EC (n=70), with four TCGA molecular types.

| Category | Total Number of cases | Pole Mutant | MSI-H Type | Low CN Type | High CN Type | χ² value | *P* value |
| --- | --- | --- | --- | --- | --- | --- | --- |
|  |  |  |  |  |  |  |  |
| **Post-menopausal** | 52 | 4 | 5 | 34 | 10 | 1.987 | 0.624 |
| **Clinical manifestations** |  |  |  |  |  |  |  |
| Abnormal uterine bleeding | 61 | 3 | 9 | 40 | 9 | 4.254 | 0.145 |
| Abnormal imaging manifestations | 66 | 3 | 9 | 43 | 11 | 4.254 | 0.145 |
| Vaginal drainage | 6 | 1 | 3 | 1 | 1 | 4.058 | 0.173 |
| Cervicitis | 6 | 0 | 1 | 5 | 0 | 1.507 | 0.740 |
| Hyperlipidemia | 5 | 0 | 0 | 3 | 2 | 1.991 | 0.494 |
| **Complications** |  |  |  |  |  |  |  |
| Atherosclerosis | 6 | 0 | 0 | 4 | 2 | 1.553 | 0.745 |
| Hypertension | 34 | 2 | 5 | 21 | 6 | 0.822 | 0.902 |
| Diabetes | 19 | 0 | 3 | 13 | 3 | 1.107 | 0.838 |
| Uterine Leiomyoma | 16 | 1 | 2 | 13 | 0 | 5.576 | 0.102 |
| Latent syphilis | 2 | 0 | 1 | 1 | 0 | 3.392 | 0.351 |
| Human Papillomavirus Infection | 1 |  |  |  | 1 | 5.223 | 0.356 |
| **Miscellaneous** |  |  |  |  |  |  |  |
| **BMI** |  |  |  |  |  | 5.513 | 0.123 |
| <28 | 43 | 3 | 7 | 23 | 10 |  |  |
| ≥28 | 27 | 0 | 2 | 22 | 3 |  |  |
| **Operation Mode** |  |  |  |  |  | 5.999 | 0.103 |
| Laparotomy | 22 | 0 | 4 | 17 | 1 |  |  |
| Laparoscope | 48 | 3 | 5 | 28 | 12 |  |  |
| **Therapy** |  |  |  |  |  |  |  |
| Adjuvant Chemotherapy | 25 | 1 | 2 | 17 | 5 | 0.962 | 0.884 |
| No therapy | 45 | 2 | 7 | 28 | 8 |  |  |
| **CEA levels** | 70 |  |  |  |  | 1.680 | 0.713 |
| Positive | 9 | 1 | 1 | 6 | 1 |  |  |
| Negative | 61 | 3 | 9 | 39 | 10 |  |  |
| **Family Genetic History of Tumor** | 9 | 0 | 1 | 7 | 1 | 0.674 | 0.903 |
| **Ca12-5** | 64 |  |  |  |  | 2.626 | 0.451 |
| Positive | 45 | 1 | 5 | 31 | 8 |  |  |
| Negative | 19 | 2 | 3 | 11 | 3 |  |  |
| **Ca15-3** | 41 |  |  |  |  | 3.055 | 1.000 |
| Positive | 1 | 0 | 0 | 1 | 0 |  |  |
| Negative | 40 | 2 | 3 | 29 | 6 |  |  |
| **Ca19-9** | 47 |  |  |  |  | 0.600 | 0.957 |
| Positive | 15 | 1 | 3 | 8 | 3 |  |  |
| Negative | 32 | 2 | 5 | 19 | 6 |  |  |

Table S3. Comparison of differing pathological types in endometrial cancer (EC) patients, with four TCGA molecular types.

| category | Total number of cases | Pole mutant | MSI-H type | Low CN type | High CN type | χ² value | P value |
| --- | --- | --- | --- | --- | --- | --- | --- |
| Pathological type | 70 |  |  |  |  | 12.156 | 0.003 |
| Type I EC | 64 | 3 | 9 | 44 | 8 |  |  |
| Type II EC | 6 | 0 | 0 | 1 | 5 |  |  |

Table S4. Comparison of differing pathological grades in endometrial cancer (EC) patients, with four TCGA molecular types.

| category | Total number of cases | Pole mutant | MSI-H type | Low CN type | High CN type | χ² value | P value |
| --- | --- | --- | --- | --- | --- | --- | --- |
| Pathological grading | 69 |  |  |  |  | 11.098 | 0.006 |
| G1~G2 | 60 | 1 | 8 | 43 | 8 |  |  |
| G3 | 9 | 1 | 1 | 2 | 5 |  |  |

Table S5. Comparison of differing tumor areas in endometrial cancer patients, with four TCGA molecular types.

| category | Total number of cases | Pole mutant | MSI-H type | Low CN type | High CN type | χ² value | *P* value |  |
| --- | --- | --- | --- | --- | --- | --- | --- | --- |
| Maximum Diameter of Tumor | 62 |  |  |  |  | 0.536 | 1.000 |  |
| ≤2cm | 21 | 1 | 2 | 16 | 5 |  |  |  |
| >2cm | 41 | 2 | 4 | 21 | 6 |  |  |  |
| Lymph Node Metastasis | 65 |  |  |  |  | 1.272 | 1.000 | |
| Positive | 3 | 0 | 0 | 3 | 0 |  |  | |
| Negative | 67 | 3 | 9 | 43 | 12 |  |  | |

Table S6. Comparison of differing vascular invasion and depth of invasion parameters in endometrial cancer patients, with four TCGA molecular types.

| Category | Total number of cases | Pole mutant | MSI-H type | Low CN type | High CN type | χ² value | P value |
| --- | --- | --- | --- | --- | --- | --- | --- |
| vascular invasion | 58 |  |  |  |  | 1.499 | 0.742 |
| positive | 13 | 1 | 2 | 8 | 2 |  |  |
| negative | 45 | 1 | 7 | 30 | 7 |  |  |
| Myometrial infiltration depth | 68 |  |  |  |  | 3.715 | 0.277 |
| No muscle layer infiltration and infiltration of muscle layer ≤ 1 / 2 | 51 | 1 | 7 | 35 | 8 |  |  |
| Infiltrating muscle layer > 1 / 2 | 17 | 2 | 2 | 9 | 4 |  |  |


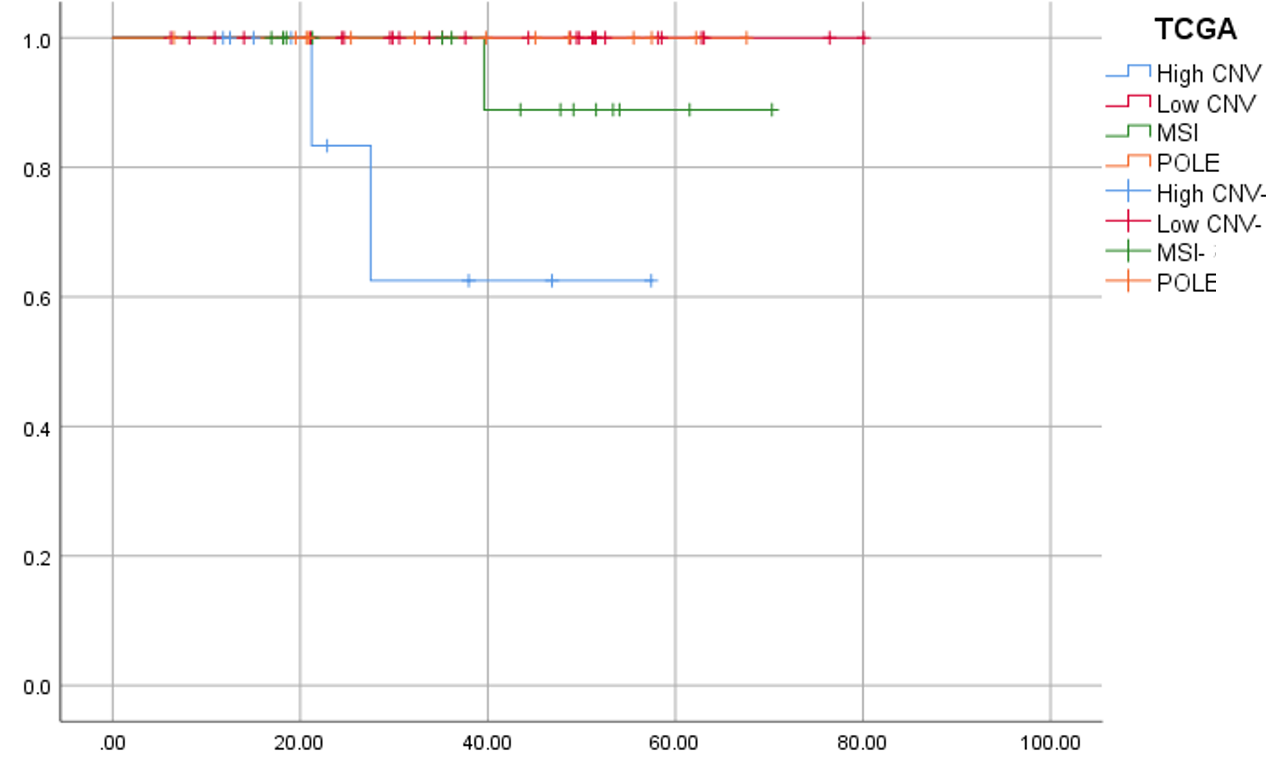


Figure S1. Overall survival curve of endometrial cancer patients, according to four TCGA molecular types.


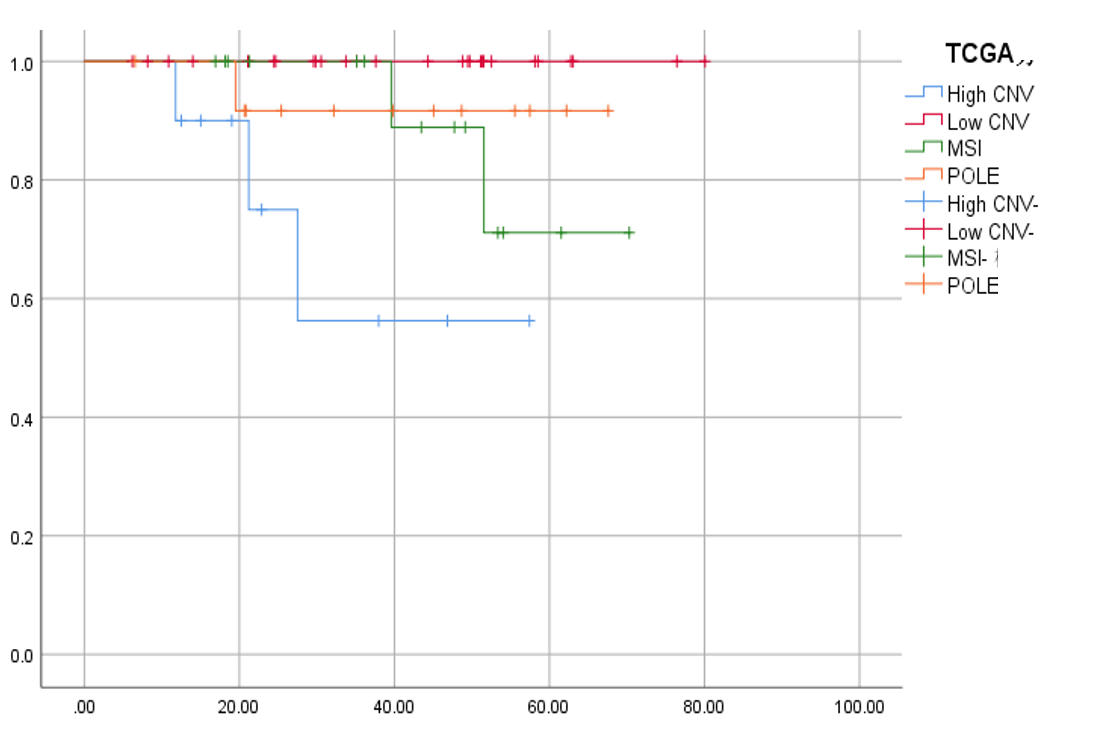


Figure S2. Progression free survival (PFS) curve of endometrial cancer patients, according to four TCGA molecular types.


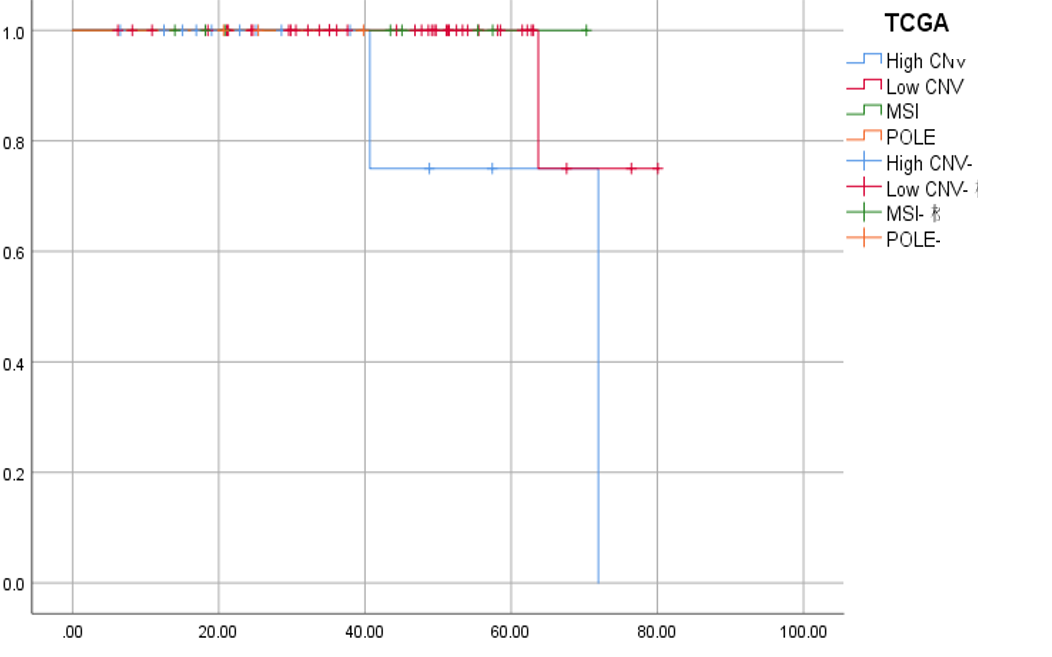


Figure S3. Overall survival (OS) curve of endometrial cancer (EC) patients, according to four TCGA molecular types


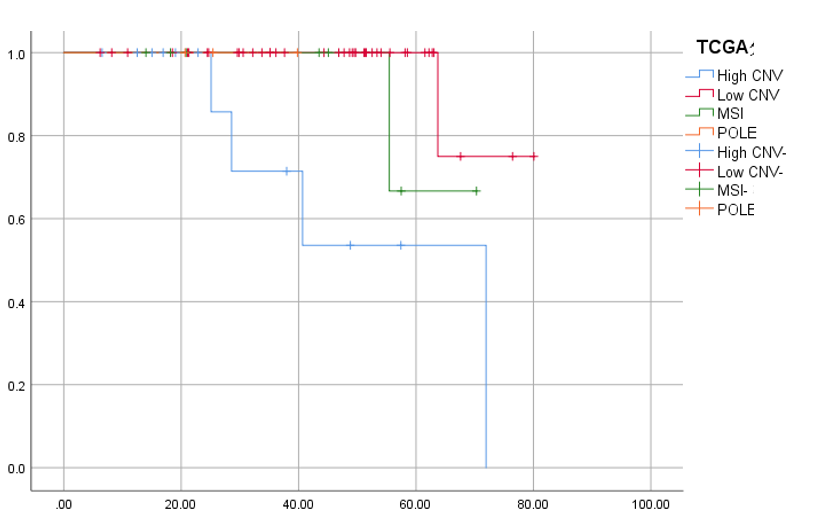


Figure S4. Progression free survival (PFS) curve of endometrial cancer (EC) patients, according to four TCGA molecular types.
